# The matrix metalloproteinase inhibitor marimastat inhibits seizures in a model of kainic acid-induced *status epilepticus*

**DOI:** 10.1038/s41598-020-78341-y

**Published:** 2020-12-04

**Authors:** Barbara Pijet, Anna Konopka, Emilia Rejmak, Marzena Stefaniuk, Danylo Khomiak, Ewa Bulska, Stanisław Pikul, Leszek Kaczmarek

**Affiliations:** 1 Laboratory of Neurobiology, BRAINCITY, Nencki Institute of Experimental Biology, Polish Academy of Sciences, 3 Pasteur Street, 02-093 Warsaw, Poland; 2 Faculty of Chemistry, Biological and Chemical Research Centre, University of Warsaw, Żwirki i Wigury 101, 02-093 Warsaw, Poland; 3 Pikralida Sp. z o.o., Bukowska 70/b424, 60-812 Poznań, Poland

**Keywords:** Blood-brain barrier, Diseases of the nervous system, Synaptic plasticity

## Abstract

An intra-hippocampus injection of kainic acid serves as a model of *status epilepticus* and the subsequent development of temporal lobe epilepsy. Matrix metalloproteinase-9 (MMP-9) is an enzyme that controls remodeling of the extracellular milieu under physiological and pathological conditions. In response to brain insult, MMP-9 contributes to pathological synaptic plasticity that may play a role in the progression of an epileptic condition. Marimastat is a metalloproteinase inhibitor that was tested in clinical trials of cancer. The present study assessed whether marimastat can impair the development of epilepsy. The inhibitory efficacy of marimastat was initially tested in neuronal cultures in vitro. As a marker substrate, we used nectin-3. Next, we investigated the blood–brain barrier penetration of marimastat using mass spectrometry and evaluated the therapeutic potential of marimastat against seizure outcomes. We found that marimastat inhibited the cleavage of nectin-3 in hippocampal neuronal cell cultures. Marimastat penetrated the blood–brain barrier and exerted an inhibitory effect on metalloproteinase activity in the brain. Finally, marimastat decreased some seizure parameters, such as seizure score and number, but did not directly affect *status epilepticus*. The long-term effects of marimastat were evident up to 6 weeks after kainic acid administration, in which marimastat still inhibited seizure duration.

## Introduction

Epilepsy is a brain disorder that is characterized by an enduring predisposition to generate epileptic seizures. At the neuroanatomical level, epilepsy is manifested by structural changes, such as neuronal reorganization, including axonal sprouting that is especially prominent in the hippocampus^1^. Epilepsy is not a homogeneous disorder but rather a collection of subtypes with various etiologies^2^. Among these etiologies, acquired epilepsies constitute approximately 60% of all cases of epilepsy and are most commonly caused by stroke, brain trauma, alcohol use, neurodegenerative diseases, and infection^3–5^. Importantly, the development of epilepsy is often a major, enduring, and debilitating condition that results from these insults. Unfortunately, approximately 30% of patients continue to experience seizures even with appropriate anti-seizure medications^6^.

In acquired epilepsies, brain-damaging insult leads to a epileptogenic latency period that lasts up to several years, culminating in the appearance of the first spontaneous seizures and an epilepsy diagnosis. Epileptogenesis is a promising therapeutic time window to apply treatments for the disease. The molecular mechanisms of epileptogenesis are under intense scrutiny. An intra-hippocampus injection of kainic acid (KA), an analog of l-glutamate and agonist of ionotropic kainate and α-amino-3-hydroxy-5-methyl-4-isoxazolepropionic acid (AMPA) receptors, induces *status epilepticus* followed by epilepsy development^7–11^.

Recently, matrix metalloproteinase-9 (MMP-9), an extracellular and extrasynaptic enzyme, has emerged as major player in synaptic plasticity^20^. MMP-9 is one of more than 20 MMPs that are calcium-dependent zinc-containing endopeptidases that can remodel the extracellular matrix (ECM) and cleave other proteins, including cell adhesion molecules, growth factors, cytokines, and cell surface receptors. Matrix metalloproteinases have been targets for drug development because of (*i*) their higher activity in numerous pathological processes, most notably tumors, (*ii*) enzymatic function that is susceptible to inhibition, and (*iii*) extracellular mode of action, making them relatively accessible to potential drugs (e.g., MMP inhibitors). Several MMP inhibitors have been developed and clinically tested but have failed in clinical trials because of their side effects with chronic treatment^12–16^.

The link between MMP-9 and epileptogenesis is supported by several lines of evidence. This enzyme is often excessively produced and released, within minutes to hours, in response to such epileptogenic insults as traumatic brain injury and *status epilepticus* that is induced by KA^17,18^. Furthermore, aberrant synaptic plasticity has been implicated in the pathogenesis of epilepsy^19^. The role of MMP-9 in synaptic plasticity has been well documented^17,20^. Several studies reported the functional involvement of MMP-9 in epileptogenesis. For example, Wilczynski et al.^21^ provided genetic evidence that MMP-9 plays a pivotal role in epileptogenesis, in which MMP-9 knockout mice exhibited a delay in epilepsy development, whereas rats that overexpressed MMP-9 solely in neurons were more prone to develop seizures. Similarly, Pijet et al.^22^ recently reported that the development of epilepsy following traumatic brain injury in mice was impaired in MMP-9 knockout mice, the number of seizures in MMP-9 KO mice after traumatic brain injury was significantly reduced and limited to 1–2 of seizures per month. While in MMP-9-overexpressing mice epilepsy development was augmented. The study also showed that increases in MMP-9 activity peaked 6–24 h after traumatic brain injury insult.

Marimastat is a broad-spectrum matrix inhibitor of several MMPs, including MMP-9^13^. Marimastat was the first MMP inhibitor that was tested in clinical trials^23^ and shown to impair tumor progression in murine cancer models^13^. It was subsequently used in human patients with pancreatic, lung, breast, colorectal, and gastric adenocarcinoma cancer^24–30^. It was shown to have a favorable pharmacokinetic profile in humans with oral administration^31^. Importantly, it was well tolerated by patients with short-term treatment. However, longer or more chronic treatment was associated with side effects that eventually precluded its use in cancer treatment^27,32–34^.

The present study investigated whether marimastat impairs seizures in an animal model. We evaluated its blood–brain barrier (BBB) permeability. We also tested whether it inhibits the cleavage of its protein substrate, nectin-3, which is considered to be MMP-9-dependent, to demonstrate the efficacy of marimastat in inhibiting the enzyme in the brain. Finally, using a mouse model of *status epilepticus* that was produced by an intra-hippocampal injection of the glutamate analog KA, we tested whether marimastat can modulate seizure episodes and seizure intensity. Although no marimastat side effects have been recognized in animal models, to provide a translational value to our study and thus to avoid potential side effects that have been described with chronic treatment in humans, we administered marimastat only acutely.

## Results

### Marimastat inhibits MMP-9 activity in vitro

**Figure 1:**
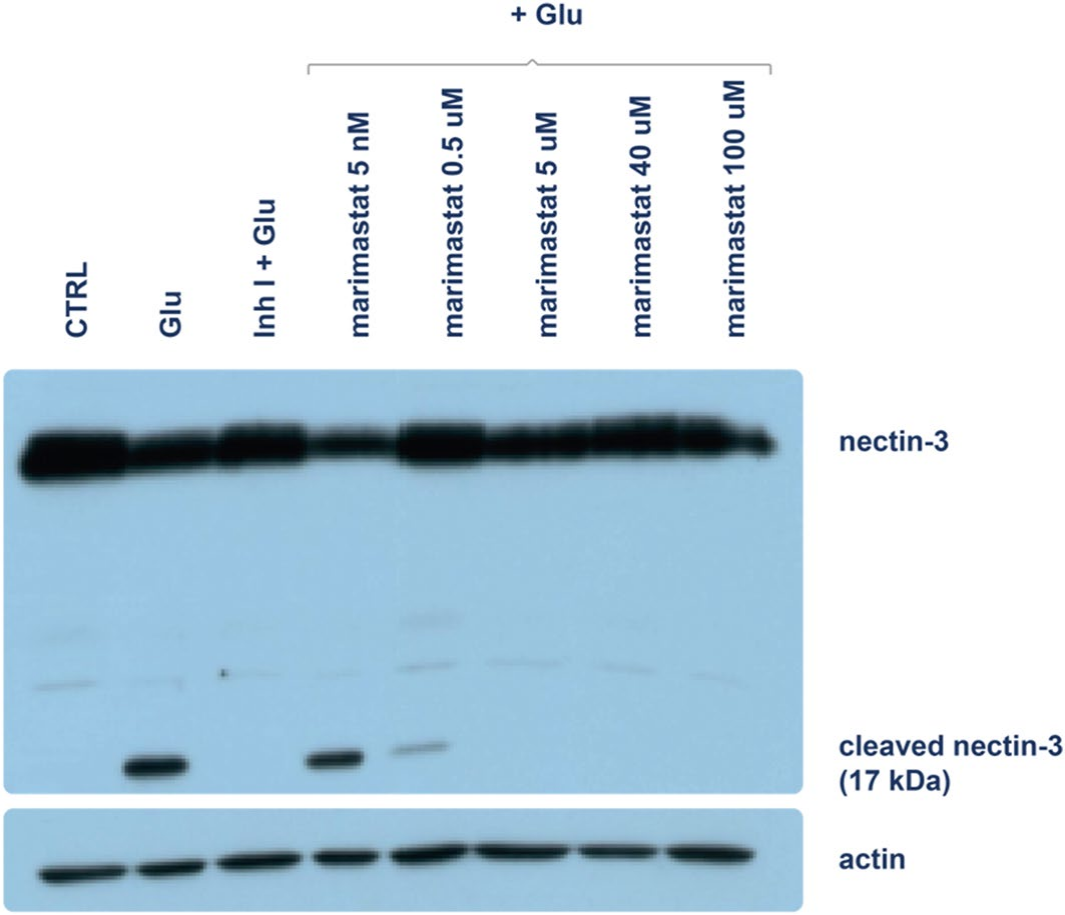
Marimastat inhibits the MMP-9-dependent cleavage of nectin-3 in hippocampal neuronal cultures. Hippocampal neurons at DIV 7 were stimulated with glutamate (Glu; 50 µM, 30 min) in the presence or absence of inhibitors (marimastat and inhibitor I). The cleavage of nectin-3 (83 kDa) results in the accumulation of a small 17 kDa proteolytic fragment. Incubation of the hippocampal neurons with marimastat abolished glutamate-induced nectin-3 cleavage.

We first tested the inhibitory effects of marimastat in hippocampal neurons that were cultured in vitro on 7 days in vitro (DIV 7). To determine the minimum effective dose, marimastat was used at the following concentrations: 5 nM, 0.5 μM, 5 μM, 40 μM, and 100 μM. Cells were incubated with marimastat for 30 min in medium supplementation, and then the cultures were treated with glutamate to stimulate neuronal cell activity, leading to the release of MMP-9. Control cultures were treated with either glutamate or glutamate in the presence of MMP-9 inhibitor I (see “Materials and Methods”). MMP-9 activity was assessed by cleavage of the MMP-9 substrate nectin-3^37^. The partial degradation of nectin-3 occurs through proteolytic shedding of the extracellular N-terminal domain and subsequent cleavage of the intracellular domain. We evaluated the basal level of nectin-3 by immunoblotting and the presence of the cleaved fragment (~ 17 kDa) of this protein. As a reference protein, we used β-actin. We found that marimastat inhibited the MMP-9-dependent cleavage of nectin-3 at 0.5 μM, and nectin-3 cleavage was completely inhibited by 5 μM marimastat (Fig. 1).

### Marimastat penetrates the blood–brain barrier

**Figure 2:**
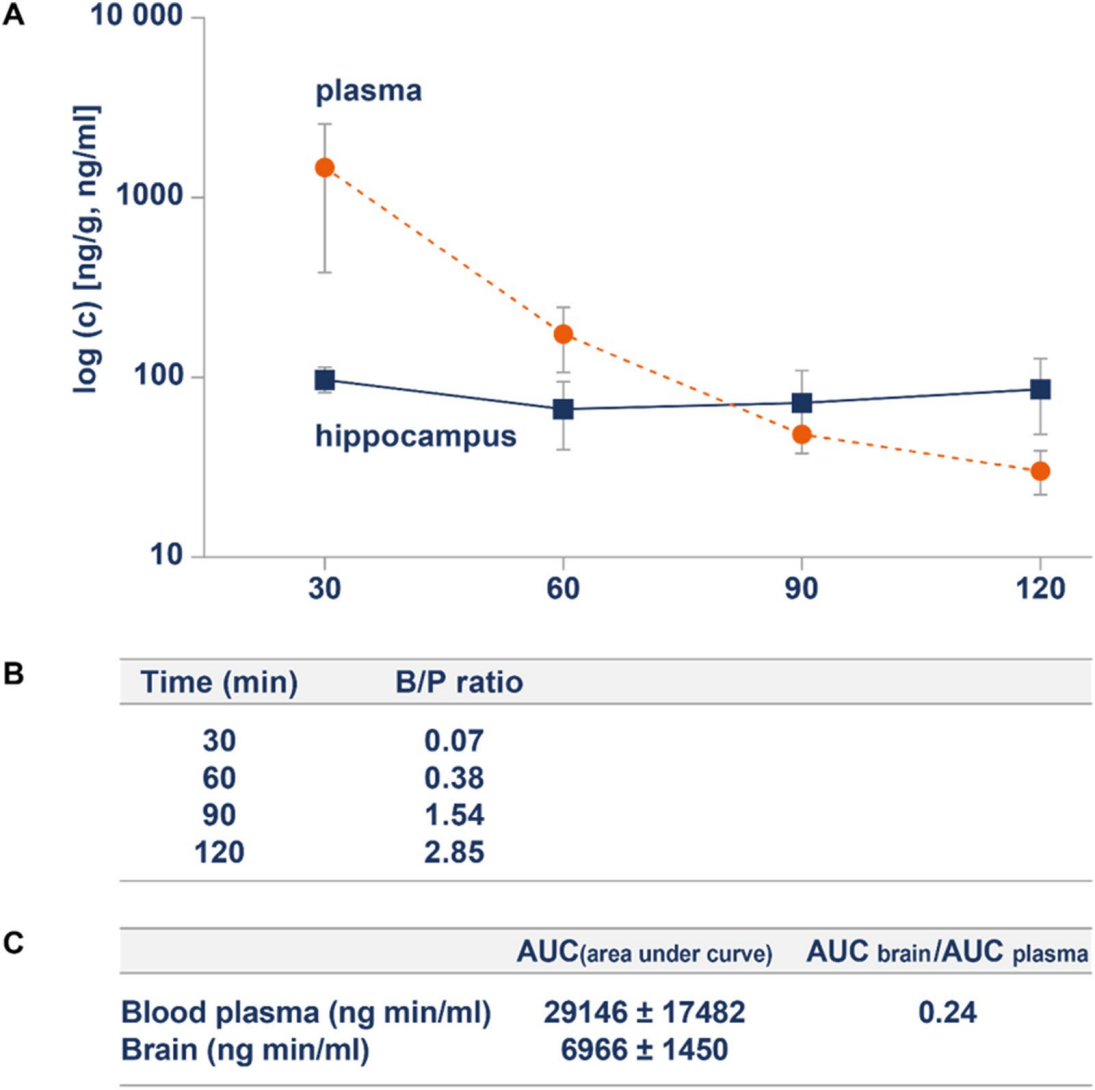
Marimastat penetrates the blood–brain-barrier. The animals were injected intraperitoneally with 9 mg marimastat; n = 3. (**A**) Measurements of marimastat concentrations in hippocampus extracts (dashed line, ng/ml) and blood plasma samples (solid line, ng/g) were performed using HPLC–ESI–MS/MS. (**B**) Brain and blood plasma marimastat concentration ratios (B/P) determined for different post-injection time points. (**C**) Areas under the kinetic curve for marimastat concentrations in hippocampal and blood plasma extracts and brain-to-plasma ratio determined for marimastat.

We initially tried to test BBB penetration with a low marimastat dose (3 mg/kg b.w.) but did not detect the compound in the brain at this dose. However, we found that marimastat was detectable in the brain tissue at 9 mg/kg b.w. Therefore, we decided to administer, in the following experiments, the drug at this concentration (intraperitoneally) three times during the first 24 h after the KA treatment. Blood plasma and hippocampus samples were collected 30, 60, 90, or 120 min after the injection. We performed quantitative analyses using high-performance liquid chromatography coupled to electrospray ionization tandem mass spectrometry (HPLC–ESI–MS/MS) in multiple reaction monitoring (MRM) mode. Marimastat was detected in both plasma and the brain (Fig. 2a). Initially high concentrations of marimastat in plasma at 30 min rapidly decreased over time, whereas its brain concentration remained constant at an average level of 80 ng/g. The brain-to-plasma (B/P) was calculated two ways. First, the B/P ratio was calculated for each post-injection time point separately as the ratio of the marimastat concentration in hippocampal extracts to blood plasma concentration (Fig. 2b). Notably, the B/P ratios for the 90 and 120 min time points exceeded 1 (1.53 and 2.85, respectively), demonstrating higher brain marimastat concentrations than plasma concentrations. Second, the ratio of areas under the kinetic curve (AUC_brain_/AUC_plasma_) of the marimastat concentration in blood plasma and hippocampal tissue was also calculated (Fig. 2c). This ratio was 0.24, which also demonstrated the ability of marimastat to efficiently penetrate the BBB.

### Marimastat blocks the MMP-9-dependent cleavage of nectin-3 in vivo

**Figure 3:**
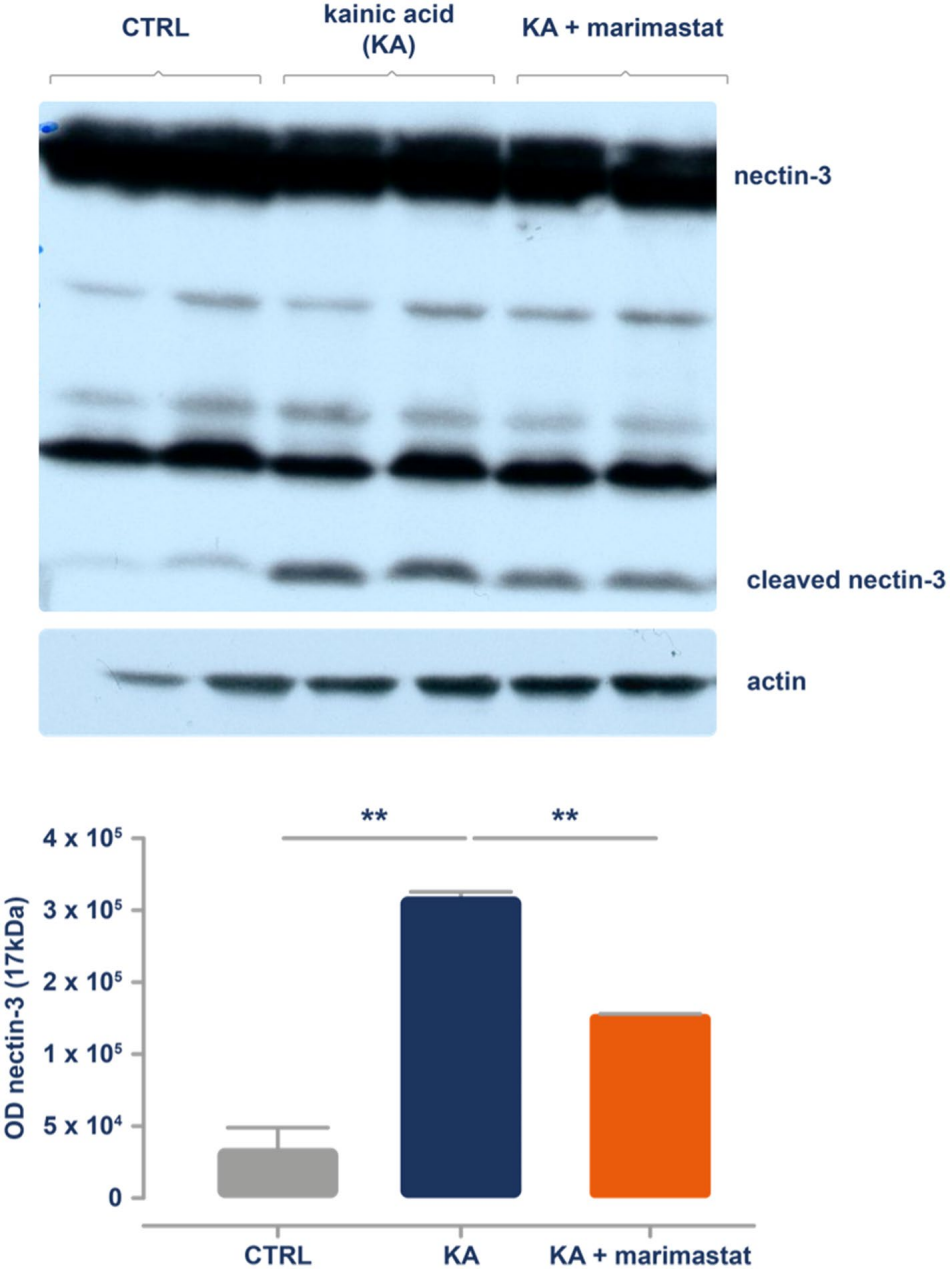
Marimastat inhibits the MMP-9-dependent cleavage of nectin-3 in a mouse model of epileptogenesis. The expression of nectin-3 (72 kDa) and the cleaved form of nectin-3 (17 kDa) was determined in mice that were injected with marimastat (9 mg/kg) intraperitoneally 1 h before administration of the pro-convulsive agent kainic acid (KA); n = 2. The injection of KA increased the cleavage of nectin-3 in the hippocampus (**p = 0.0071), and marimastat inhibited this effect (**p = 0.0022, unpaired *t*-test). The data are expressed as mean ± SEM. For detailed description please refer to Supplementary Fig. 2. Additional bands observed on western blots have been reported previously^37^ and we have not characterized them in the details.

To investigate the effects of marimastat in vivo, we used a pro-convulsive KA injection, a well-established mouse model of *status epilepticus* and subsequent epileptogenesis. In this model, KA is administered intraperitoneally, which leads to seizures that are indicative of pro-epileptogenic *status epilepticus*^38^. To evaluate the inhibitory effect of marimastat on MMP-9, the mice were injected intraperitoneally with marimastat (9 mg/kg) 1 h before KA administration. To assess whether marimastat inhibits MMP-9 in the brain, the hippocampus was isolated 6 h after the KA injection, and an immunoblotting assay was performed to detect the cleavage of nectin-3 protein. We found that marimastat administration reduced the enzymatic cleavage of nectin-3 by 66%, reflected by the optical density of the 17 kDa nectin-3 fragment (*p* = 0.0022; Fig. 3).

### Marimastat ameliorates seizures in epileptic mice

**Figure 4:**
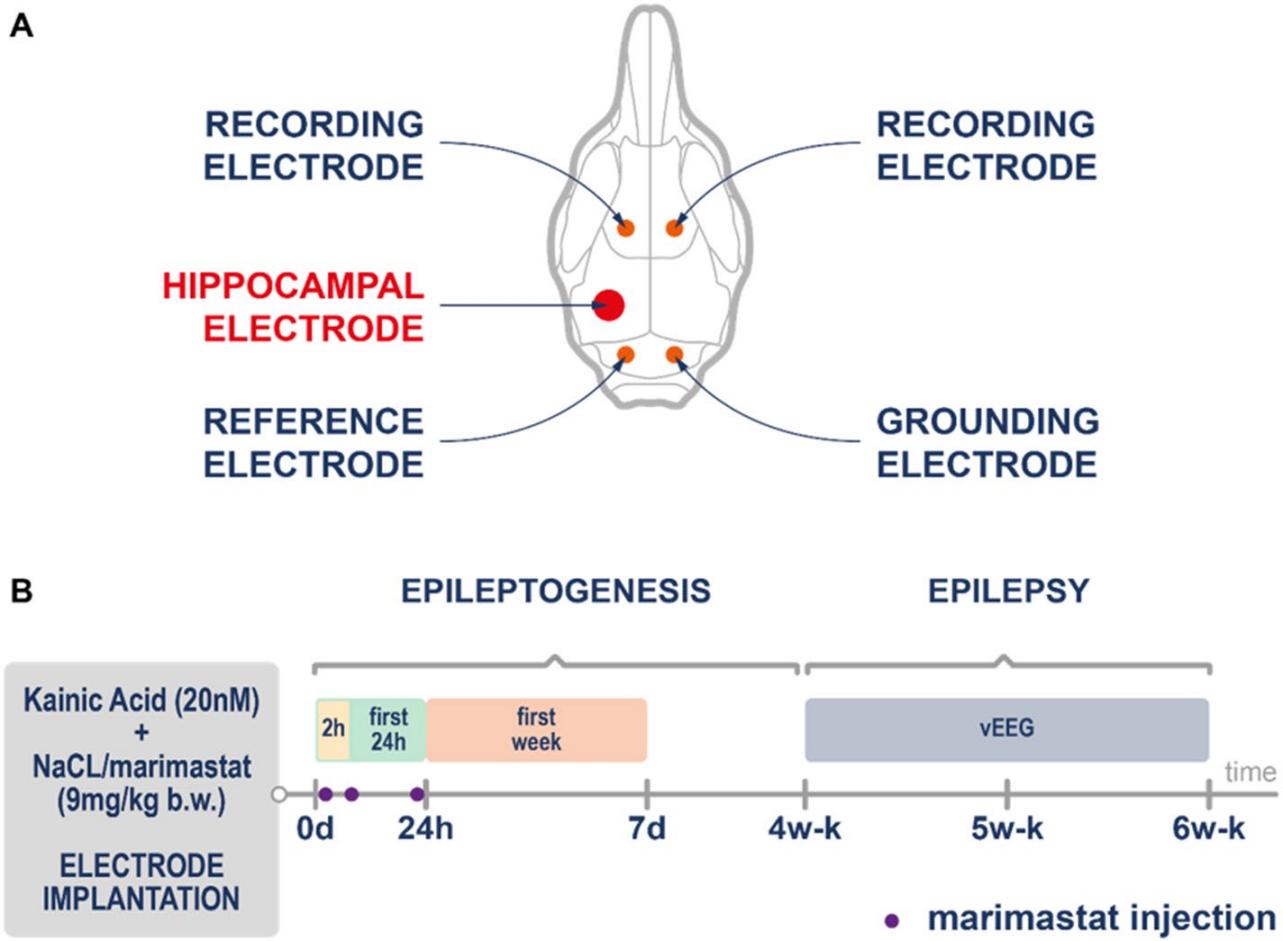
(**A**) Placement of skull and hippocampal electrodes**.** (**B**) Study design. Skull and hippocampal electrode implantation was performed together with kainic acid injections. Video EEG monitoring lasting for 1 week starting on the day of implantation. Chronic seizures were recorded for the next 4 weeks. Marimastat was injected 30 min, 6 h, and 24 h after the intra-hippocampus kainic acid injection.

The study design is presented in Fig. 4. In this part of the study, we evaluated the therapeutic effect of marimastat on KA-induced seizures. After the KA injection and electrode implantation (Fig. 4a; for details, see “Material and Methods”), the mice were placed in Plexiglas cages and connected with commutators. Marimastat was injected three times: 30 min, 6 h, and 24 h after the KA injection (Fig. 4b). Video electroencephalography (EEG) was recorded at four stages: 0–2 h, first 24 h, first week (days 2–8), and for 2 weeks between weeks 4 and 6 after the KA injection (Fig. 4b). During the first 2 h, after the KA injection, the seizure number and latency to the first seizure were assessed. At the next stages (24 h, first week, and weeks 4–6), we studied such parameters as seizure duration (in seconds), seizure severity (according to the Racine scale), and seizure number (per animal/per day). Status epilepticus was observed in mice after KA injection during first 24 h after surgery. Mortality of the animals after intra-hippocampus KA administration was around 30% in both the control and marimastat-treated groups. Setting the onset and the end-point of a single seizure was based on EEG recordings and visual inspection. To assign each seizure to relevant point on Racine scale, EEG was compared to the video recording that was registered in parallel.

**Figure 5:**
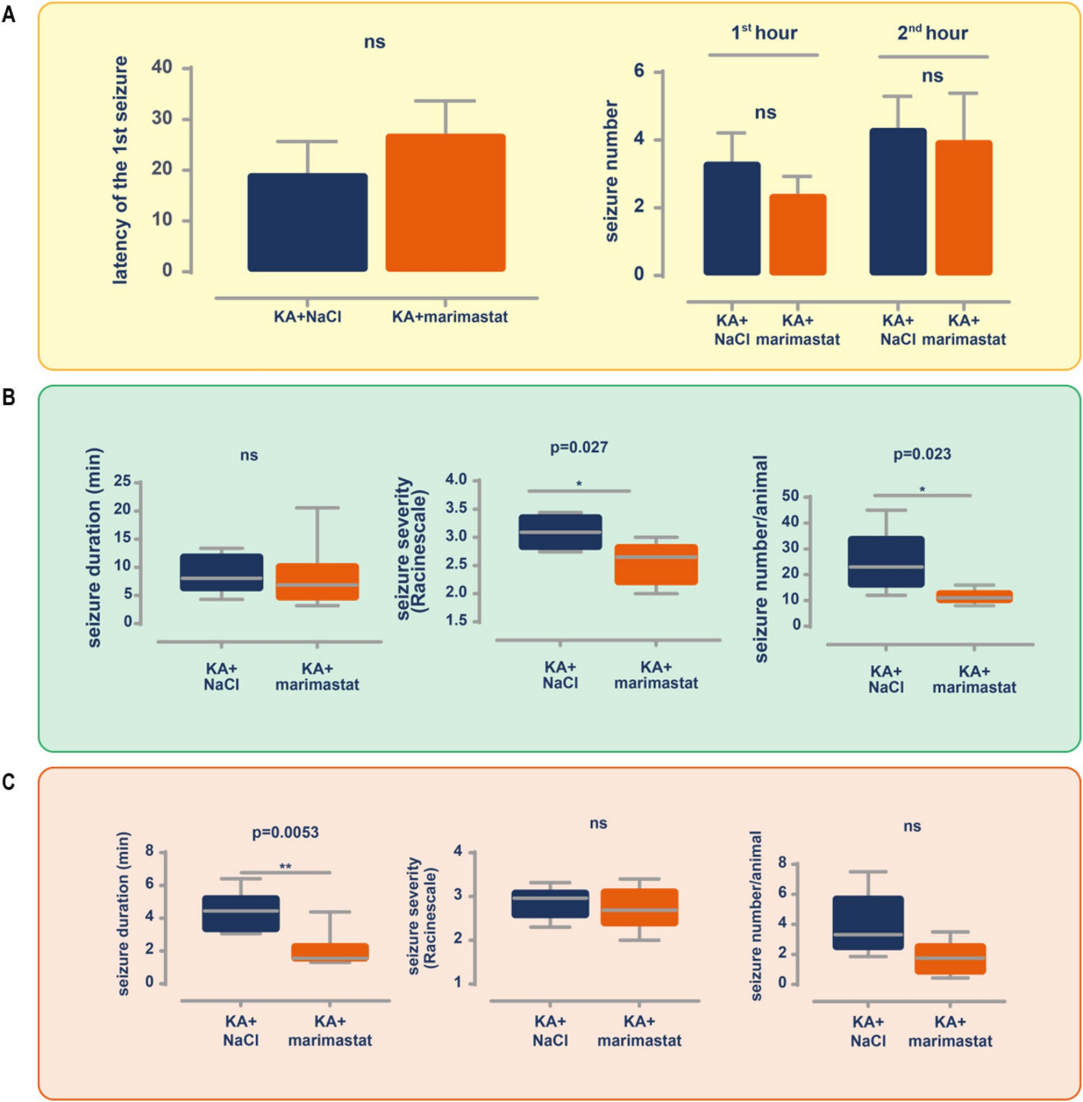
Marimastat reduces seizure appearance evoked by kainic acid during the first week after the intra-hippocampus injection**.** (**A**) No effect of marimastat on *status epilepticus* evoked by kainic acid during the first 2 h post-injection. (**B**) Marimastat reduced seizure severity and seizure number evoked by kainic acid during the first 24 h post-injection. (**C**) Marimastat reduced seizure duration evoked by kainic acid during the first week post-injection. Seizure duration indicates the average. Seizure severity was determined according to the modified Racine scale. The seizure number was measured per animal per 24 h of video EEG recording. The data are expressed as mean ± SEM. *p < 0.05, **p < 0.001 (unequal variance *t*-test [Welch’s *t*-test]); n = 4.

During the first 2 h after KA administration, we observed no effect of marimastat on seizure number (hour 1, *p* > 0.05; hour 2, *p* > 0.05) or the latency to the first seizure (Fig. 5a). During the first day after the KA injection, marimastat significantly decreased seizure severity (*p* = 0.0278; Fig. 5b) and seizure number during the first 24 h (*p* = 0.0238; Fig. 5b), with no effect on seizure duration (*p* = 0.3506; Fig. 5b).

**Figure 6:**
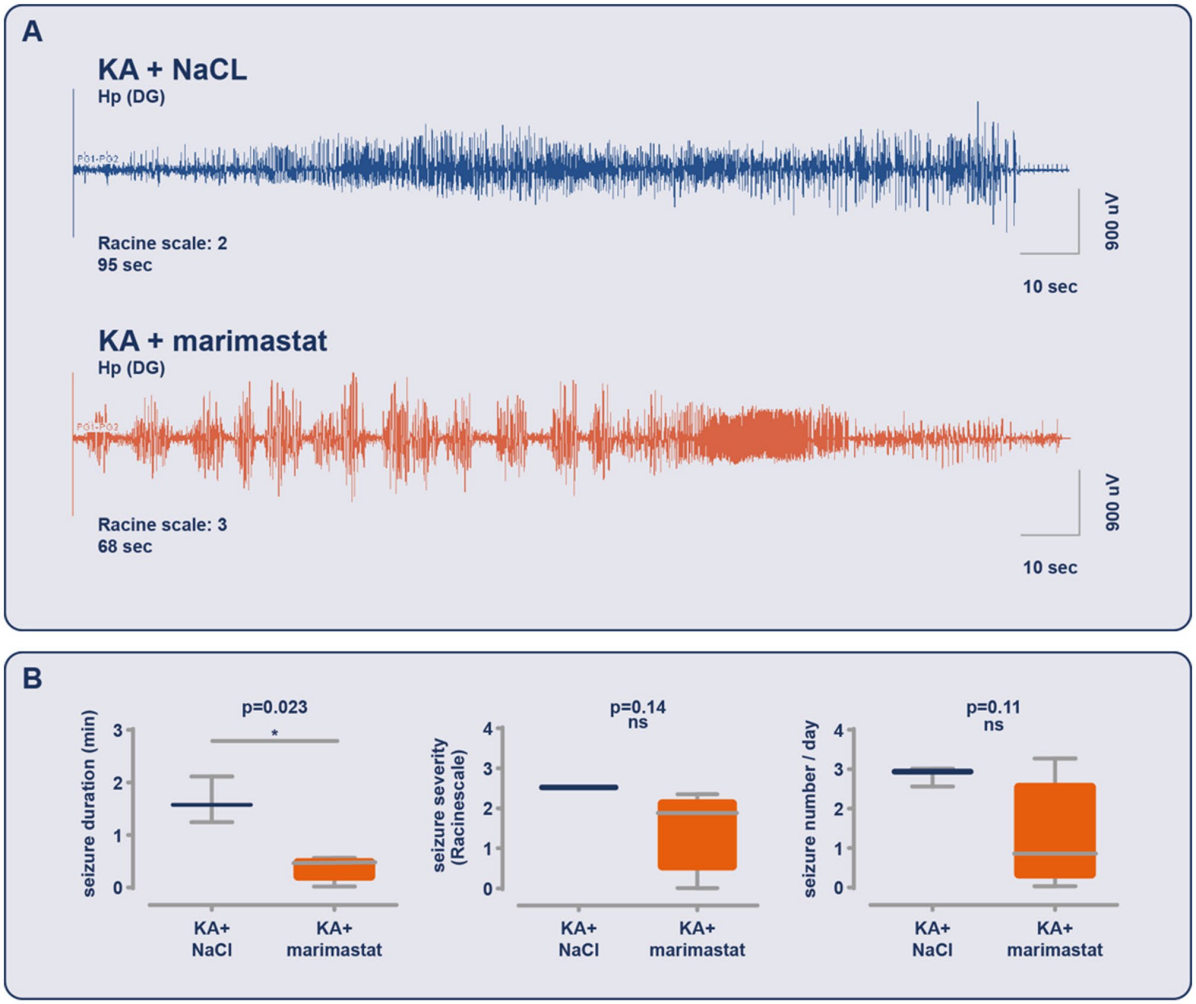
Marimastat reduces the duration of chronic seizures evoked by kainic acid. Chronic seizures monitoring was performed between 4 and 6th day after KA injection. (**A**) An example seizure was recorded by a bipolar hippocampal electrode (blue: control group injected with saline + KA; orange: marimastat group injected with marimastat + KA). (**B**) Seizure duration but not seizure severity and number per day were altered following marimastat treatment. Seizure duration is the average. Seizure severity was determined according to the modified Racine scale. The seizure number was measured per animal per 24 h of video EEG recording. The data are expressed as mean ± SEM. *p < 0.05, **p < 0.001 (unequal variance *t*-test [Welch’s *t*-test]); n = 4.

In the second part of the experiment, we analyzed data from the next 7 days of EEG recording with regard to the appearance of spontaneous seizures using the same parameters. In contrast to the first 24 h, during the next week of observation, we found a difference only in seizure duration, in which marimastat shortened the duration of a single seizure (*p* = 0.0053; Fig. 5c). Seizure severity and number did not change (*p* = 0.6143 and *p* = 0.0900, respectively; Fig. 5c). To further analyze the therapeutic effect of marimastat on the appearance and parameters of seizures, we also evaluated chronic changes. We evaluated seizure appearance, duration, number, and severity (Fig. 6b). One month after epileptogenesis induction, marimastat inhibited the duration of a single seizure (*p* = 0.023; Fig. 6a,b). Moreover, marimastat slightly decreased seizure score and number, but these changes did not reach statistical significance (*p* = 0.1494 and *p* = 0.1108, respectively; Fig. 6).

## Discussion

In the present study, marimastat potently inhibited MMP-9 activity that was evoked by glutamate in hippocampal cultures in vitro, in which it prevented proteolytic cleavage of the MMP-9 substrate nectin-3. Marimastat effectively penetrated the BBB and accumulated in the brain after peripheral administration, demonstrated by MS. Marimastat also inhibited proconvulsive KA-driven MMP-9 activity in the brain, reflected by a decrease in the proteolytic cleavage of nectin-3. Finally, marimastat impaired the development of seizures that were evoked by KA-driven *status epilepticus*.

The major goal of the present study was to assess whether marimastat is useful for impairing seizure outcomes that are evoked by KA-induced *status epilepticus*. Epilepsy is a major, long-lasting, and debilitating complication following various brain insults. The latent period of epileptogenesis that might be supported by aberrant synaptic plasticity is believed to be responsible for the development of epilepsy. MMP-9 has been repeatedly shown to play a pivotal role in both physiological and aberrant synaptic plasticity and shown to contribute to epileptogenesis, in which it is rapidly and transiently activated by pro-epileptogenic insults and functionally involved in epilepsy development^17,36^. Marimastat has been previously tested in clinical trials for the prevention of tumor metastases, but it failed in the trials because chronic treatment resulted in side effects^36^. Therefore, we reasoned that the acute and time-limited administration of marimastat following pro-epileptogenic *status epilepticus* might be beneficial for preventing epilepsy development [see also^36^].

In the present study, we first found that marimastat inhibited MMP-9 activity in neurons. Marimastat was previously shown to be a broad-spectrum MMP inhibitor and active against MMP-9, but this notion was not apparently tested in neurons. We also sought to validate inhibitor efficacy by testing substrate cleavage in the brain. A limited number of neuronal MMP-9 substrates have been identified to date. Among these, the partial proteolytic cleavage of β-dystroglycan was previously used as an indicator of MMP-9 activity^39^. Unfortunately, the appropriate antibody for β-dystroglycan is no longer available. Therefore, we chose another MMP-9 substrate, nectin-3^37^. Glutamate treatment, which is known to activate MMP-9, provoked the partial proteolysis of nectin-3, releasing the 17 kDa fragment that was visible by Western blot. The observed kinetics of production of the cleavage product was consistent with the dynamics of MMP-9 activation^37^, and marimastat prevented this cleavage.

In previous studies and clinical trials, marimastat was used to block MMP activity in peripheral tissues, although it was also shown to affect gliomas in the brain^40^. However, no rigorous investigations of its permeability of the BBB to reach the brain have been conducted. Thus, we used HPLC–ESI–MS/MS to measure marimastat concentrations in blood plasma and brain tissue at different post-injection time points. HPLC–ESI–MS/MS is currently the most frequently used analytical method in pharmacokinetic studies that seek to quantify the concentration of chemical substances in different tissues^41,42^, including BBB permeability studies^43^. We recently applied this analytical technique to examine the BBB penetration of a novel compound that inhibits gelatinase activity^44^. The AUC_brain_/AUC_plasma_ ratio is a useful parameter for assessing the BBB permeability of a compound^45^. Compounds that are characterized by a AUC_brain_/AUC_plasma_ ratio greater than 0.1 are considered to have sufficient access to the brain. The AUC_brain_/AUC_plasma_ ratio for marimastat was 0.24, indicating its BBB permeability. The B/P ratio from the pharmacokinetic study was calculated separately for each post-injection time point. In a previous study, diazepam, which is considered to be a highly brain-penetrant compound, B/P ratios are in the range of 2.6–4.0 within the time period of 15–120 min after administration^44^. Diazepam rapidly penetrates the BBB, but it is also quickly removed from both blood and the brain. In contrast, in the present study, plasma marimastat concentrations decreased over time but remained at a constant level in the brain. The following B/P ratios for marimastat were obtained: 0.07, 0.38, 1.54, and 2.85 at 30, 60, 90, and 120 min, respectively. Overall, we found that marimastat efficiently penetrated into the brain following the intraperitoneal injection and preferentially accumulated in brain tissue. In this context, it might be arguable that marimastat accumulates around the neurovasculature, with only limited penetration into brain parenchyma. However, such a phenomenon would not explain nectin-3 cleavage because this protein has been detected in parenchyma.

To test whether marimastat inhibits MMP-9 activity in the brain, we injected KA peripherally in mice. KA was previously shown to markedly activate MMP-9^18,21,45^. Furthermore, KA administration serves as a well-described model of *status epilepticus*-driven epileptogenesis. In the present study, KA administration produced nectin-3 cleavage, indicating an increase in MMP-9 activity, which was diminished by marimastat treatment. This result strongly suggested that marimastat effects were mediated by its ability to inhibit MMP-9. We cannot, however, exclude other marimastat targets. The direct testing of this notion by use of the drug in MMP-9 KO mice, subjected to KA treatment, is not workable, since only a very low number of MMP-9 KO mice undergo epileptogenesis^22^.

The less effective inhibition of nectin-3 cleavage in the brain than in neuronal cultures might be attributable to the complexity of the brain’s response to KA, the pharmacokinetics of the penetration of marimastat into the brain, and the relatively low dose of marimastat that was tested, which was used to avoid potential side effects.

To reveal the whole spectrum of marimastat activity against the development of epilepsy, we determined behavioral seizure scores and various EEG features, such as seizure duration, seizure number, and seizure severity. Marimastat was effective against seizure severity and number during first 24 h after the KA injection, whereas marimastat inhibited seizure duration within the next 7 days and weeks.

In conclusion, the present study found that marimastat, a drug that was previously tested in human clinical trials for other disease conditions, may be useful for impairing the development of epileptogenesis and as an adjunct therapy for seizures. Despite the growing interest in addressing epileptogenesis to prevent the full-blown clinical manifestation of the disease, no anti-epileptogenic drug has yet emerged. Furthermore, we found a possible molecular mechanism of action of marimastat, in which it inhibited MMP-9 in the brain that was exposed to glutamate receptor agonist-induced neuronal excitation.

## Materials and methods

### Animals

The experiments were performed on adult (12–14-week-old) male C57BL/6J mice (Jackson Laboratory, Bar Harbor, ME, USA). The animals were housed in individual cages (22 °C ± 1 °C, 50–60% humidity, food and water free access), under a 12 h/12 h light/dark cycle. All of the procedures were performed in accordance with the Animal Protection Act of Poland (directive 2010/63/EU) and were approved by the First Warsaw Local Ethics Committee for Animal Experimentation (permission no. 609/2014).

### Marimastat

Marimastat was purchased from Sigma-Aldrich (St. Louis, MO, USA; catalog no. M2699) as a colorless powder with an HPLC purity > 98%. For the in vitro and in vivo experiments, marimastat was dissolved in saline. For the in vivo experiments, the mice were injected with marimastat intraperitoneally. Because of its side effects after prolonged use in cancer preclinical trials, the marimastat dose was reduced to 9 mg/kg^23,24,27,32–34^. To detect the MMP-9-dependent cleavage of nectin-3 in vivo and in vitro and BBB permeability, marimastat was administered in a single dose. To evaluate the inhibitory effect on KA-induced seizures, marimastat was injected in three repeated doses for the first 24 h after KA administration^31^.

### MMP-9 dependent cleavage of nectin-3 in vitro

Hippocampal neurons were prepared from Wistar rats (P0). Plates were covered (5% PDL, poly-D-lysine; Sigma) and washed (sterile Milli-Q water). Hippocampi were isolated on ice and placed in dissociated medium (DM; 1 M Na_2_SO_4_, 0.5 M K_2_SO_4_, 1 M MgCl_2_, 100 mM CaCl_2_, 1 M HEPES, 2.5 M glucose, 0.5% Phenol Red, 0.1 M NaOH). The cultures were incubated in papain solution (Cell Systems) (30 min, 37 °C). Tissue was next washed with DM and incubated with a trypsin inhibitor (Sigma) for 10 min (37 °C). Next, the cultures were washed with MEM (Modified Eagle Medium) (Gibco), homogenized with fresh MEM, and placed on plates (3 × 10^6^ cells/plate). After 2 h, MEM was removed and replaced with Neurobasal supplemented with: B27, 1 mM l-glutamine, 100 U/ml penicillin, 0.1 mg/ml streptomycin (Invitrogen). On DIV 7, the cultures were incubated with marimastat (30 min; 5 nM, 0.5 µM, 5 µM, 40 µM, 100 µM) inhibitor I (5 µM; Merck, #444,252) and stimulated with glutamate (30 min; 50 μM). Inhibitor I is piperazine-based, a cell-permeable and highly, potent inhibitor of MMP-9 and MMP-13 (IC50 = 900 pM) but at much higher concentrations it also inhibits MMP-1, MMP-3 and MMP-7. As a control, non-stimulated neurons and cells that were stimulated only with glutamate were used. After stimulation, the cells were lysed (4 × sample buffer with 2-mercaptoethanol). The samples were incubated for 6 min (96 °C). Next, proteins were separated by electrophoresis, and Western blot analysis was performed. For analysis the same volume of each sample was used.

### MMP-9 dependent cleavage of nectin-3 in vivo

For the experiments that evaluated the influence of marimastat on the MMP-9-dependent cleavage of nectin-3, the animals were injected with marimastat. Because of the fact that apparently, no antibody available that recognizes only the cleaved form of nectin-3 (17 kDA) in the brain tissue sections, nectin-3 cleavage was evaluated with use of western blot analysis, as described previously^37^. One hour later, KA was injected intraperitoneally (40 mM), and the mice were observed for the next 6 h for the seizures appearance. Next, the hippocampus was dissected and stored at − 80 °C. After tissue was thawed, the samples were homogenized in buffer that contained 10 mM CaCl_2_, 0.25% Triton X-100, protease inhibitor cocktail (cOmplete Mini EDTA-free; Roche) and centrifuged (6000×*g*, 30 min, 4 °C). The supernatant was recovered. The pellet (Triton X-100-insoluble) was re-suspended in a buffer that contained 50 mM Tris (pH 7.4) and 0.1 M CaCl_2_ in water, incubated for 15 min (60 °C), and centrifuged (10,000×*g*, 30 min, 4 °C). This treatment results in the release of ECM-bound MMPs into the solution. The final supernatant was considered a Triton X-100-insoluble fraction. After centrifugation, the supernatant was recovered. Sample protein concentrations were measured using the BCA protein assay (Pierce, Rockford, USA).

### Western blot

SDS-PAGE was performed (150 V, RT) and proteins were transferred to polyvinylidene difluoride membranes (200 mA). The membranes were blocked (1 h, RT), incubated with primary antibody (nectin-3: 1:1000, catalog no. ab63931, Abcam; β-actin: 1:5000, catalog no. A1978, Sigma) overnight (4 °C), washed 3 × in Tris-buffered saline/0.1% Tween-20 (TBS-T; 10 min, shaking) and incubated with secondary HRP-conjugated antibodies (in blocking buffer, 1 h, RT; anti-rabbit P-100, 1:5000; anti-mouse P-200, 1:5000; Vector Laboratories). After TBS-T washing, the signal was developed (ECL Western Blotting Detection Reagent; GE Healthcare) and detected on Medical X-ray Blue/MXBE Film (Carestream) using a Fuji Film Processor (FPM-800A). The optical density of the nectin-3/actin bands was quantified (GeneTool program).

### Blood–brain barrier permeability

To evaluate the BBB permeability of marimastat, mice were injected intraperitoneally with marimastat, as described previously^44^. Thirty, 60, 90, or 120 min post-injection, the mice were anesthetized (sodium pentobarbital, 150 mg/kg), and venous blood was collected. The animals were perfused with ice-cold PBS, after which hippocampus tissue samples were collected (n = 5). Blood was centrifuged at 14,000×*g* for 30 min at RT in the presence of sodium citrate. The brain samples were weighed and homogenized in 500 ml of deionized water. Marimastat was extracted using acetonitrile/water (50/50, v/v, LC/MS grade, JT Baker). 100 µl and 50 µl of hippocampus and plasma samples were added to 500 µl and 250 µl of extraction buffer, respectively. The samples were incubated for 10 min, RT, 800 rpm. After centrifugation (17,500×*g*, RT, 10 min) pellets were discarded, and supernatants were transferred to LC autosampler vials. The HPLC–ESI–MS/MS analysis was performed using an Agilent 1290 Infinity LC system coupled to an Agilent 6460 Triple Quadrupole Mass Spectrometer equipped with an ESI source operating in negative polarity. The mass spectrometer was operated in MRM mode following the optimization of working conditions for marimastat using a standard solution at 1 mg/l. The following m/z transitions were monitored: 330.1 → 279.1, 330.1 → 241.0, 330.1 → 222.1, 330.1 → 169.1, and 330.1 → 142.1. Compounds were separated on a Zorbax Eclipse Plus C18 Rapid Resolution column (Agilent Technologies; 100 mm × 4.6 mm, 3.5 µm) at 35 °C. Water with 0.1% formic acid and acetonitrile with 0.1% formic acid were used as eluents A and B, respectively. The mobile phase was delivered at 0.5 ml/min in isocratic mode with 50% of eluent B. The injection volume was 10 µl. The quantification of marimastat was achieved by the external matrix calibration curve method. Blank extracts were obtained from untreated animals following the same procedure. The calibration curve for brain aliquots was generated within the concentration range of (0.338–6.75) ng/ml (R^2^ = 1). For plasma samples, the calibration curve was generated within two concentration ranges: (6.75–67.5) ng/ml (R^2^ = 0.999) and (13.5–135) ng/ml (R^2^ = 0.991). Considering the dilution factor and masses of tissues collected, the content of marimastat was calculated per gram or milliliter, respectively. The limit of quantification of marimastat (the lowest point of the calibration curve) was (31.64 ± 1.58) ng/g in brain samples and (20.24 ± 0.89) ng/ml in plasma samples.

### Intracranial electrode implantation and electroencephalographic recording

The study design is presented in Fig. 4b. For the experiments that evaluated the inhibitory effects of marimastat on nectin-3 and seizures, the mice were divided into two groups. Each group received an intra-hippocampus injection of KA (20 mM in 0.9% NaCl). Thirty minutes, 6 h, and 24 h after the KA injection, the mice were injected intraperitoneally with saline/marimastat (n = 6). The animals were anesthetized (domitor 0.5 g/kg; ketamine 8 g/kg, i.p.) and placed in a stereotaxic frame on a heating pad. The mice were injected with 70 nl of KA solution (flow rate: 50 nl/min) in the left CA1 field of the dorsal hippocampus at the following coordinates: anterior/posterior, − 1.8 mm; medial/lateral, + 1.7 mm, dorsal/ventral, − 2.3 mm from bregma. Immediately after the KA injection, four stainless-steel screw electrodes were placed in the skull (∅ 1.6 mm, Bilaney Consultants). Additionally, one bipolar hippocampal electrode (Bilaney Consultants) was placed in the injected hippocampus at the following coordinates: anterior/posterior, − 2.0 mm; medial/lateral, + 1.3 mm from bregma; dorsal/ventral, − 1.7 mm below dura. Cortical recording electrodes were placed bilaterally in the skull: two over the frontal cortex, two (reference electrode and ground electrode) over the cerebellum (Fig. 4a). After surgery, the mice were placed in Plexiglas cages (one mouse/cage) and connected to the recording system with commutators (SL6C, Plastics One, Roanoke, VA, USA).

Video EEG was performed using the Twin EEG recording system that was connected to a Comet EEG PLUS with a 57-channel AS40-PLUS amplifier (Natus Medical) and filtered (high-pass filter cut-off 0.3 Hz, low-pass filter cut-off 100 Hz). Video EEG activity was monitored during: the first 2 h after KA administration, the first 24 h, the next 7 days, at weeks 4–6 for 2 weeks (Fig. 4b). The occurrence of seizures was evaluated by visual inspection of the EEG and video recordings. The following parameters were analyzed: seizure number, duration, severity, spontaneous seizure number. Spontaneous seizures were defined as seizures that appeared as early as 24 h after KA administration. Seizure severity was estimated based on the modified Racine scale^35^. An EEG seizure was defined as a high-amplitude (> 2 × baseline) rhythmic discharge that clearly represented an abnormal EEG pattern that lasted > 5 s. The frequency of seizures was calculated as the number of seizures per day/week. The EEG analysis was performed by visual inspection of trained experimenter. Blinding was applied only at the last stage of experiment (EEG recordings analysis).

### Statistical analyses

The results are expressed as mean ± SEM. All of the analyses were conducted using Prism 7.02 software (GraphPad, La Jolla, CA, USA). Differences between experimental groups were considered significant if the type 1 error was less than 5%.

## Supplementary Information

Supplementary Information.

## References

[1] Pitkänen A, Lukasiuk K, Dudek FE, Staley KJ (2015). Epileptogenesis. Cold Spring Harb. Perspect..

[2] Sirven JI (2015). Epilepsy: a spectrum disorder. Cold Spring Harb. Perspect. Med..

[3] Annegers JF, Hauser WA, Coan SP, Rocca WA (1998). A population-based study of seizures after traumatic brain injuries. N. Engl. J. Med..

[4] Graham NSN, Crichton S, Koutroumanidis M, Wolfe CDA, Rudd AG (2013). Incidence and associations of poststroke epilepsy: the prospective South London Stroke Register. Stroke.

[5] Hesdorffer DC, Logroscino G, Cascino G, Annegers JF, Hauser WA (1998). Risk of unprovoked seizure after acute symptomatic seizure: effect of *status epilepticus*. Ann. Neurol..

[6] Pitkänen A, Lukasiuk K (2011). Mechanisms of epileptogenesis and potential treatment targets. Lancet Neurol..

[7] Arabadzisz D, Antal K, Parpan F, Emri Z, Fritschy JM (2005). Epileptogenesis and chronic seizures in a mouse model of temporal lobe epilepsy are associated with distinct EEG patterns and selective neurochemical alterations in the contralateral hippocampus. Exp. Neurol..

[8] Carriero G (2012). A guinea pig model of mesial temporal lobe epilepsy following nonconvulsive *status epilepticus* induced by unilateral intrahippocampal injection of kainic acid. Epilepsia.

[9] Nadler JV, Cuthbertson GJ (1980). Kainic acid neurotoxicity toward hippocampal formation: dependence on specific excitatory pathways. Brain. Res..

[10] Daniels WM, Jaffer A, Engelbrecht AH, Russell VA, Taljaard JJ (1990). The effect of intrahippocampal injection of kainic acid on corticosterone release in rats. Neurochem. Res..

[11] Raedt R (2009). Seizures in the intrahippocampal kainic acid epilepsy model: characterization using long-term video-EEG monitoring in the rat. Acta Neurol. Scand..

[12] Winer A, Adams S, Mignatti P (2018). Matrix metalloproteinase inhibitors in cancer therapy: turning past failures into future successes. Mol. Cancer Ther..

[13] Rasmussen HS, McCann PP (1997). Matrix metalloproteinase inhibition as a novel anticancer strategy: a review with special focus on batimastat and marimastat. Pharmacol. Ther..

[14] Parsons SL, Watson SA, Steele RJ (1997). Phase I/II trial of batimasat, a matrix metalloproteinase inhibitor, in patients with malignant ascities. Eur. J. Surg. Oncol..

[15] Macaulay VM (1999). Phase I study of intraplural batimastat (BB-94), a matrix metalloproteinase inhibitor, in the treatment of malignant pleural effusions. Clin. Cancer Res..

[16] Vandenbroucke RE, Libert C (2014). Is there new hope for therapeutic matrix metalloproteinase inhibition?. Nat. Rev. Drug Discov..

[17] Beroun A (2019). MMPs in learning and memory and neuropsychiatric disorders. Cell Mol. Life Sci..

[18] Szklarczyk A, Lapinska J, Rylski M, McKay RDG, Kaczmarek L (2002). Matrix metalloproteinase-9 undergoes expression and activation during dendritic remodeling in adult hippocampus. J. Neurosci..

[19] Pitkänen A, Engel J (2011). Past and present definitions of epileptogenesis and its biomarkers. Neurotherapeutics.

[20] Vafadari B, Salamian A, Kaczmarek L (2016). MMP-9 in translation: from molecule to brain physiology, pathology, and therapy. J. Neurochem..

[21] Wilczynski GM (2008). Important role of matrix metalloproteinase 9 in epileptogenesis. J. Cell Biol..

[22] Pijet B (2018). Elevation of MMP-9 levels promotes epileptogenesis after traumatic brain injury. Mol. Neurobiol..

[23] Steward WP, Thomas AL (2000). Marimastat: the clinical development of a matrix metalloproteinase inhibitor. Expert Opin. Investig. Drugs.

[24] Bramhall SR (2002). Marimastat as maintenance therapy for patients with advanced gastric cancer: a randomised trial. Br. J. Cancer.

[25] Evans JD (2001). A phase II trial of marimastat in advanced pancreatic cancer. Br. J. Cancer.

[26] Rosemurgy A (1999). Marimastat in patients with advanced pancreatic cancer: a dose-finding study. Am. J. Clin. Oncol..

[27] Wojtowicz-Praga S (1998). Phase I trial of marimastat, a novel matrix metalloproteinase inhibitor, administered orally to patients with advanced lung cancer. J. Clin. Oncol..

[28] Miller KD (2002). A randomized phase II pilot trial of adjuvant marimastat in patients with early-stage breast cancer. Ann. Oncol..

[29] North H, King J, Morris DL (2000). Effect of marimastat on serum tumour markers in patients with colorectal cancer. Int. J. Surg. Investig..

[30] Kimata M (2002). Matrix metalloproteinase inhibitor, marimastat, decreases peritoneal spread of gastric carcinoma in nude mice. Jpn. J. Cancer Res..

[31] Millar AW (1998). Results of single and repeat dose studies of the oral matrix metalloproteinase inhibitor marimastat in healthy male volunteers. Br. J. Clin. Pharmacol..

[32] King J, Zhao J, Clingan P, Morris D (2003). Randomised double blind placebo control study of adjuvant treatment with the metalloproteinase inhibitor, marimastat in patients with inoperable colorectal hepatic metastases: significant survival advantage in patients with musculoskeletal side-effects. Anticancer Res..

[33] Renkiewicz R (2003). Broad-spectrum matrix metalloproteinase inhibitor marimastat-induced musculoskeletal side effects in rats. Arthritis Rheum..

[34] Sparano JA (2004). Randomized phase III trial of marimastat versus placebo in patients with metastatic breast cancer who have responding or stable disease after first-line chemotherapy: Eastern Cooperative Oncology Group trial E2196. J. Clin. Oncol..

[35] Racine RJ (1972). Modification of seizure activity by electrical stimulation: II. Motor seizure. Electroencephalogr. Clin. Neurophysiol..

[36] Ikonomidou C (2014). Matrix metalloproteinases and epileptogenesis. Mol. Cell. Pediatr..

[37] van der Kooij MA (2014). Role for MMP-9 in stress-induced downregulation of nectin-3 in hippocampal CA1 and associated behavioural alterations. Nat. Commun..

[38] Lévesque M, Avoli M, Bernard C (2016). Animal models of temporal lobe epilepsy following systemic chemoconvulsant administration. J. Neurosci. Methods.

[39] Michaluk P (2007). β-dystroglycan as a target for MMP-9, in response to enhanced neuronal activity. J. Biol. Chem..

[40] Groves MD (2002). Phase II trial of temozolomide plus the matrix metalloproteinase inhibitor, marimastat, in recurrent and progressive glioblastoma multiforme. J. Clin. Oncol..

[41] Pitt JJ (2009). Principles and applications of liquid chromatography-mass spectrometry in clinical biochemistry. Clin. Biochem. Rev..

[42] Wong AL (2018). A review on liquid chromatography-tandem mass spectrometry methods for rapid quantification of oncology drugs. Pharmaceutics.

[43] Reichel A (2009). Addressing central nervous system (CNS) penetration in drug discovery: basics and implications of the evolving new concept. Chem. Biodivers..

[44] Bertran A (2020). Design and synthesis of selective and blood-brain barrier-permeable hydroxamate-based gelatinase inhibitors. Bioorg. Chem..

[45] Fenyk M (2004). Comparison of the effects of perfusion in determining brain penetration (brain-to-plasma ratios) of small molecules in rats. Comp. Med..

