# Supplementary material for: The matrix metalloproteinase inhibitor marimastat inhibits seizures in a model of kainic acid-induced status epilepticus
Source: Sci Rep. 2020 Dec 4;10:21314. doi: 10.1038/s41598-020-78341-y (PMC7718901; doi:10.1038/s41598-020-78341-y)
Supplement: Supplementary file 1 — Supplementary Information. [file 41598_2020_78341_MOESM1_ESM.pdf]

Suppplementary file

The matrix metalloproteinase inhibitor marimastat inhibits seizures in a model of kainic acid-induced *status epilepticus*

Barbara Pijet,[1]* Anna Konopka,[2] Emilia Rejmak,[1] Marzena Stefaniuk,[1] Danylo Khomiak,[1] Ewa Bulska,[2] Stanisław Pikul,[3] Leszek Kaczmarek[1]

[1] Laboratory of Neurobiology, BRAINCITY, Nencki Institute of Experimental Biology, Polish Academy of Sciences, 3 Pasteur Street, Warsaw, 02-093, Poland
[2] University of Warsaw, Faculty of Chemistry, Biological and Chemical Research Centre, Żwirki i Wigury 101, Warsaw, 02-093, Poland
[3] Pikralida sp. z o.o. Bukowska 70/b424, Poznań, 60-812, Poland

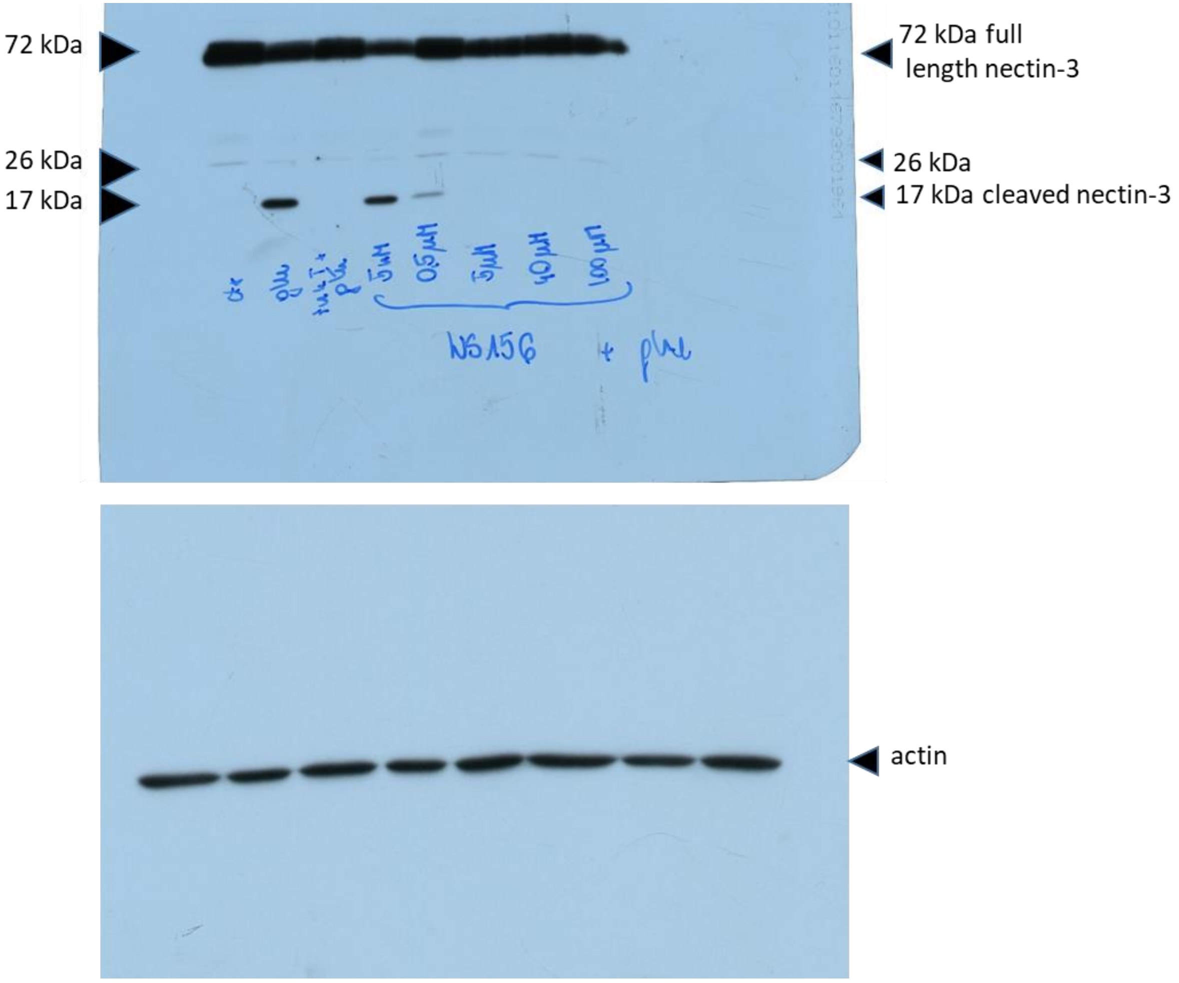

Supplementary Figure 1. Western Blots presenting the expression of full length nectin-3 and cleaved form in protein extracts from hippocampal cell cultures. Cell cultures were treated with glutamate with addition respectively: inhibitor I and different concentrations of marimastat. Lower panel represents an expression of the reference protein – actin. Figure refers to manuscript Fig. 1.

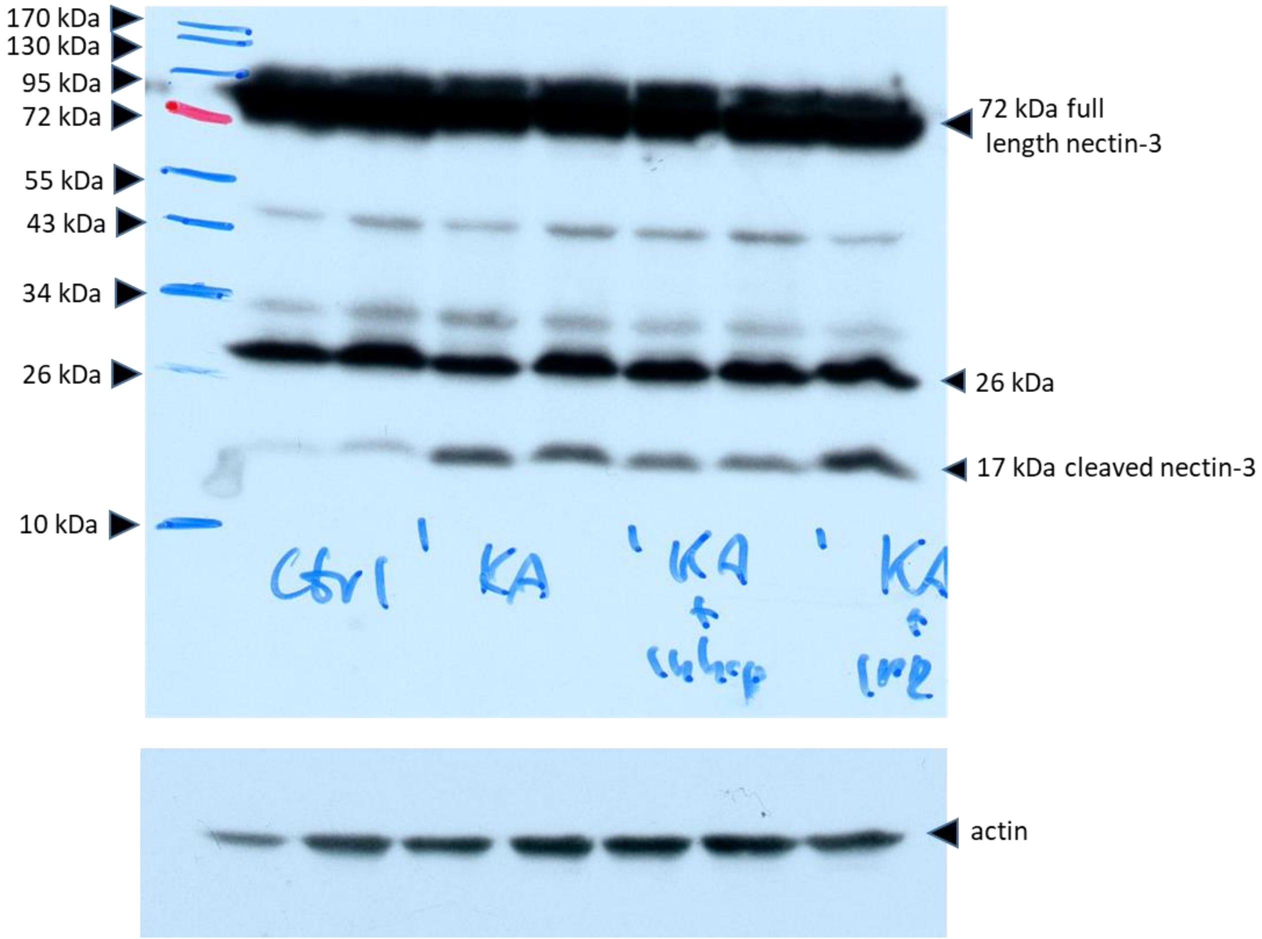

Supplementary Figure 2. Western Blots presenting the expression of full length nectin-3 and cleaved form in the hippocampus brain tissue lysates. Blots represents respectively: control mice injected with saline (1st-2nd line), mice injected intrahippocampally with kainic acid (3rd-4th line) and mice with combined injection of marimastat and KA (5th-6th line). Last line shows treatment with a different inhibitor (unsuccessful). Lower panel presents the expression of reference protein – actin. Figure refers to manuscript Fig. 3.

A

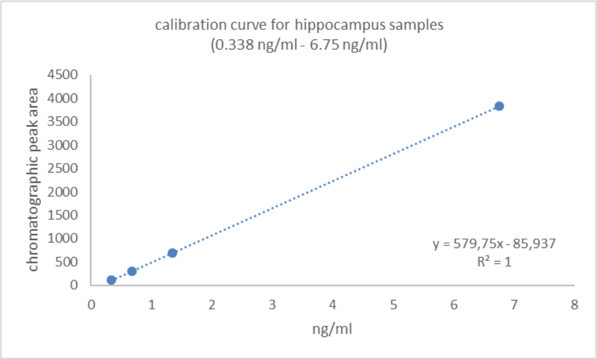

hippocampus samples			
concentration	chromatographic peak area		
ng/ml			
0.338	111	119	100
0.675	228	322	299
1.35	668	694	711
6.75	3669	3794	3862

B

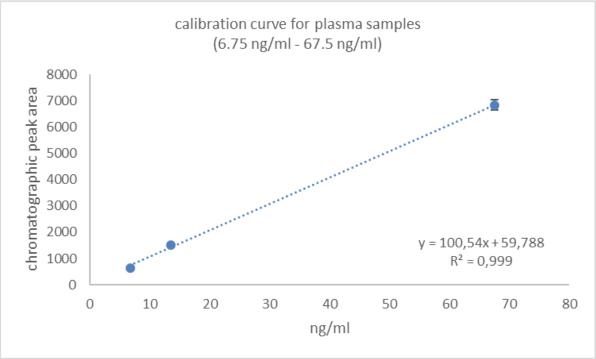

plasma samples			
concentration	chromatographic peak area		
ng/ml			
6.75	551	619	659
13.5	1417	1511	1547
67.5	6731	6698	6970

C

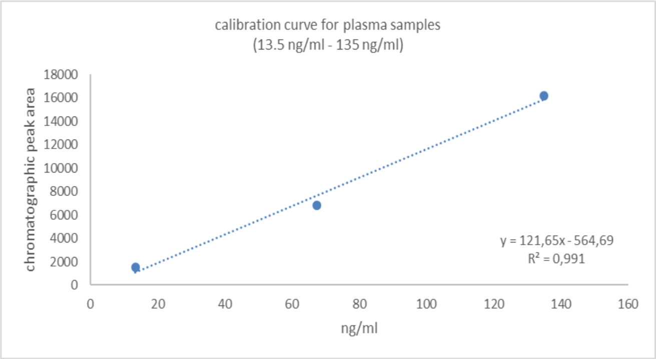

plasma samples			
concentration	chromatographic peak area		
ng/ml			
13.5	1417	1511	1547
67.5	6731	6698	6970
135	15913	16173	16265

Supplementary Figure 3. Calibration curve for hippocampus and plasma samples in the concentration range (**A** 0.338 – 6.75 for hippocampus; **B** 6.75 – 67.5 and **C** 13.5 – 135 ng/ml for plasma) and individual measurement values used for their construction. Errors bars – SD. Figure refers to manuscript Fig. 2.
